# Therapeutic Use of 3β-[N-(N′,N′-Dimethylaminoethane) Carbamoyl] Cholesterol-Modified PLGA Nanospheres as Gene Delivery Vehicles for Spinal Cord Injury

**DOI:** 10.1371/journal.pone.0147389

**Published:** 2016-01-29

**Authors:** So-Jung Gwak, Yeomin Yun, Do Heum Yoon, Keung Nyun Kim, Yoon Ha

**Affiliations:** 1 Spine & Spinal Cord Institute, Department of Neurosurgery, Yonsei University College of Medicine, Seoul, Korea; 2 Department of Bioengineering, Clemson University, Clemson, South Carolina, United States of America; The University of Tennessee Health Science Center, UNITED STATES

## Abstract

Gene delivery holds therapeutic promise for the treatment of neurological diseases and spinal cord injury. Although several studies have investigated the use of non-viral vectors, such as polyethylenimine (PEI), their clinical value is limited by their cytotoxicity. Recently, biodegradable poly (lactide-co-glycolide) (PLGA) nanospheres have been explored as non-viral vectors. Here, we show that modification of PLGA nanospheres with 3β-[N-(N′,N′-dimethylaminoethane) carbamoyl] cholesterol (DC-Chol) enhances gene transfection efficiency. PLGA/DC-Chol nanospheres encapsulating DNA were prepared using a double emulsion-solvent evaporation method. PLGA/DC-Chol nanospheres were less cytotoxic than PEI both *in vitro* and *in vivo*. DC-Chol modification improved the uptake of nanospheres, thereby increasing their transfection efficiency in mouse neural stem cells *in vitro* and rat spinal cord *in vivo*. Also, transgene expression induced by PLGA nanospheres was higher and longer-lasting than that induced by PEI. In a rat model of spinal cord injury, PLGA/DC-Chol nanospheres loaded with vascular endothelial growth factor gene increased angiogenesis at the injury site, improved tissue regeneration, and resulted in better recovery of locomotor function. These results suggest that DC-Chol-modified PLGA nanospheres could serve as therapeutic gene delivery vehicles for spinal cord injury.

## Introduction

The delivery of therapeutic genes has been developed as a potential treatment for central nervous system injury [1,2]. The use of viral vectors to deliver genes has been in experimental animal models [3,4]. However, viral vectors induce a strong immune and inflammatory response, limiting their clinical application [5,6]. In contrast to viral vectors, non-viral vectors such as copolymers and cationic lipids and liposomes are advantageous due to their low toxicity, low tendency to induce immune responses, high tissue-specific targeting, good ease and scale of production, and good handling properties [7–9]. Although non-viral vectors have been proposed as alternatives to viral vectors, there is still a need to improve their low transfection rates and gene expression efficiencies.

Several studies demonstrate that nanospheres formed from biodegradable poly (lactic-co-glycolic acid) (PLGA) copolymer are efficient vehicles for various types of therapeutic agents such as drugs and genes [7,10–14]. PLGA is biocompatible and approved by the United States Food and Drug Administration for human clinical use for a variety of applications such as surgical screws and sutures [8]. PLGA nanospheres facilitate the sustained release of encapsulated plasmid DNA (pDNA) and thus have the potential to direct long-term gene expression [12,15]. Due to their small size, PLGA nanospheres are taken up by cells through endocytosis and are capable of endo-lysomal escape. However, unmodified PLGA nanospheres have low gene transfection efficiency compared to other delivery vehicles comprised of cationic lipids or polymers [16,17]. For this reason, cationic excipients such as chitosan, polyethyleneimine (PEI), and cationic lipids and polyamines have been added to PLGA-based gene delivery systems to improve their transfection properties.

Many cationic lipids in the form of liposomes show good gene transfer activity. The positively charged lipid binds to pDNA via electrostatic forces, leading to the formation of DNA-lipid complexes that are taken up into cells by endocytosis [18]. Cationic lipids such as 3β-[N-(N′,N′-dimethylaminoethane) carbamoyl] cholesterol (DC-Chol) or dioleoylphosphatidylethanolamine (DOPE) have been extensively investigated in studies of gene delivery, with several lipids having been tested in various stages of clinical trials [19] due to their low toxicity [20]. DC-Chol possesses a tertiary amine that is not ionized at neutral to alkaline pH levels; therefore, it reduces the aggregation of DNA-liposome complexes and leads to higher transgene expression [21]. However, DC-Chol liposomes have several limitations, such as poor serum stability and short circulation *in vivo* [22,23].

In this study, we investigated whether DC-Chol-modified PLGA nanospheres enable more efficient gene delivery than unmodified PLGA nanospheres. First, we evaluated the physicochemical and cytotoxic properties of PLGA/DC-Chol nanospheres. We investigated whether PLGA/DC-Chol nanospheres enhance cellular uptake and gene expression in mouse neural stem cells and enhance gene transfection efficiency after direct injection into the rat spinal cord. Also, we evaluated the therapeutic efficacy of PLGA/DC-Chol nanospheres as delivery vehicles for the vascular endothelial growth factor (VEGF) gene in a rat model of spinal cord injury.

## Material and Methods

### Nanosphere preparation

PLGA-hybrid nanospheres encapsulating pDNA were prepared using a double emulsion-solvent evaporation method as previously described [7,16]. Briefly, 1 ml luciferase pDNA (pSV-Luc) or pSV-VEGF in Tris/EDTA buffer was emulsified in a PLGA solution (5% w/v in methylene chloride, MW = 66,000 Da; Birmingham Polymers, Birmingham, AL, USA) with or without DC-Chol solution (5% w/v in methylene chloride, Birmingham) using a sonicator for 5 min. A water-in-oil solution was emulsified in 25 ml of 4% (w/v) aqueous polyvinyl alcohol (PVA, MW = 30,000–70,000 Da; Sigma, St. Louis, Mo) solution using a sonicator for 5 min. The emulsion was stirred for 72 h at room temperature to remove methylene chloride. PLGA nanospheres were recovered by ultracentrifugation (20,000g for 20 min at 4°C). The PLGA nanosphere pellet was washed five times in distilled water to remove PVA and was then re-suspended by vortexing and lyophilizing for 48 h to obtain a dry powder.

### Nanosphere characterization

Size distribution and zeta potential of nanospheres encapsulating the pSV-Luc gene were evaluated using dynamic laser light scattering (Zetasizer 3000HS, Malvern, UK). Morphological examination of nanospheres was performed using a scanning electron microscope (SEM; JSM6330F, JEOL, Tokyo, Japan). To determine the amount of pSV-Luc incorporated into PLGA or PLGA/DC-Chol nanospheres, 10 mg of nanospheres encapsulating pSV-Luc were dissolved in 0.5 ml of 0.5M NaOH while stirring at 37°C until a clear solution was obtained [14,16]. pDNA content in the resulting solution was quantified by a UV spectrophotometer (Shimadzu 1204, Tokyo, Japan) at 260 nm by means of a standard curve of pDNA in solution of blank nanospheres dissolved NaOH.

### Structural integrity of pDNA

pDNA extracted from nanospheres was analyzed by gel electrophoresis for purity and structural integrity before and after encapsulation. pDNA was extracted from nanospheres using 0.5N NaOH. Products were analyzed using 0.8% (w/v) agarose gel electrophoresis with ethidium bromide staining. Gels were read using a gel documentation system (Gel Doc 1000, Bio-Rad Laboratories, Hercules, CA, USA).

### *In vitro* release of pDNA

To analyze the kinetics of pDNA release from PLGA or PLGA/DC-Chol nanospheres, pDNA-loaded nanospheres were suspended in 2 ml phosphate-buffered saline (PBS, pH 7.4) at 37°C under continuous agitation. The supernatant was then withdrawn, and PBS was replenished. Amount of pDNA in the supernatant was determined with a UV spectrophotometer. Experiments were performed in quintuplicate.

### *In vitro* cytotoxicity

The cytotoxicity of PLGA nanospheres, PEI (MW = 25,000 Da; Sigma) or lipofectamine (Life Technologies) was determined by measuring mitochondrial metabolic activity in cultured mouse neural stem cells (mNSCs). mNSCs were plated in 96-well plates at 2×10^4^ cells/well and cultured for 24 h. Different concentrations of PLGA/DC-Chol nanospheres or PEI encapsulating pDNA were added to each well. After incubation for 4, 24, or 48 h at 37°C, mNSCs were rinsed with PBS, and 200 μl MTT (3-(4,5-dimethylthiazol-2-yl)- 2,5-diphenyltetrazolium bromide, 2 mg/ml in PBS; Sigma) was added. After incubation for 4 h at 37°C, MTT solution was removed. The resulting insoluble purple particles were dissolved in 100 μl dimethyl sulfoxide hybrid-max (DMSO; Sigma) for 30 min, and absorbance was measured at 540 nm using an ELISA plate reader. Percentage of cell metabolic activity was calculated by normalizing absorbance values with those of mNSCs not treated with polymer.

### *In vitro* cellular uptake

To measure uptake of nanospheres by mNSCs, nanospheres containing pDNA and 50 mg fluorescent dye (6-coumarin; Polysciences, Warrington, PA, USA) were prepared [7,16]. The solution of 6-coumarin in methylene chloride was mixed with PLGA/DC-Chol solubtion. A water-in-oil solution was emulsified in 25 ml of 4% (w/v) aqueous polyvinyl alcohol (PVA) solution using a sonicator for 5 min. The emulsion was stirred for 72 h to remove methylene chloride. PLGA nanospheres were recovered by ultracentrifugation. The PLGA nanosphere pellet was washed five times in distilled water and lyophilizing for 48 h to obtain a dry powder. mNSCs were plated at 1×10^4^ cells/well and allowed to attach for 24 h, after which nanospheres containing pDNA and fluorescent dye were added. After 6 or 24 h incubation at 37°C, the medium was removed, and cells were washed with PBS. mNSCs were fixed in 4% (v/v) paraformaldehyde (PFA) at room temperature, and Vectashied mounting medium with DAPI (4,6- diamidino-2-phenylindole; Vector Laboratories) was used to stain cell nuclei. Cells transfected with nanospheres were observed using confocal laser scanning microscopy (Fluoview BX50, Olympus, Tokyo, Japan).

### *In vitro* transfection

mNSCs were plated in 6-well plates at 2×10^5^ cells/well and cultured for 24 h. pSV-Luc-encapsulating PLGA nanospheres or PEI (25,000 Da)/pSV-Luc complexes (the number of nitrogen in PEI per DNA phosphate, N/P ratio = 5/1) were suspended in DMEM/F12, added to each well at 2 μg pDNA/well, and incubated at 37°C. Afterward, the medium was changed to DMEM/F12 supplemented with 10% (v/v) fetal bovine serum. The medium was changed every 2 days. Fourteen days after transfection, 100 μl lysis buffer (Cell Culture Lysis Reagent 5×, Promega, Madison, WI, USA) was added to each well. Lysates were incubated on ice and cleared by centrifugation for 10 min at 13,000g using an ultracentrifuge. Transfection efficiency was measured using a luminometer (TD20/20, Turner Design, Sunnyvale, CA, USA). Total protein content in the supernatant was determined by a BCA protein assay kit (Pierce, Iselin, NJ). Results are expressed as relative light units (RLU)/mg protein.

### *In vivo* cytotoxicity

The spinal cord injury model involved adult male Sprague-Dawley rats (250–300 g; OrientBio Gyeonggi-do, Korea). All procedures were approved by the Animal Care and Use Committee of Yonsei University College of Medicine and were in accordance with international guidelines on the ethical use of animals. The number of animals used was minimized. PLGA nanospheres, PLGA/DC-Chol nanospheres, or PEI (25,000 Da)/pDNA complexes (N/P ratio:5/1) were injected into the spinal cord of healthy rats. One day after transplantation, rats were sacrificed and perfused with saline containing PFA (Merck, Germany). Apoptotic activity was measured by TUNEL staining using the ApopTag Plus Fluorescein In situ Apoptosis Detection kit (Chemicon International. Temecula, CA).

### *In vivo* transfection

After anesthesia with sodium penthobarbital (20 mg/kg; Choongwae Pharma, Seoul, Korea), laminectomy was performed at the T9 level. PLGA or PLGA/DC-Chol nanospheres encapsulating pSV-Luc were immediately injected using an insulin syringe into the injured epicenter of the spinal cord. In other groups of rats, PEI/pSV-Luc complex, naked pSV-Luc, or PBS was injected into the injured epicenter spinal cord. After surgery, antibiotic sepazolin (50 mg/kg, Yuhan Corporation) was administered for 7 days. Transgene expression in the spinal cord was evaluated 14 days after surgery by a luciferase assay. Rats were sacrificed and perfused with saline (pH 7.4), and the spinal cord including the epicenter was removed. Each specimen was homogenized in lysis buffer (Cell Culture Lysis Reagent 5×, Promega, Madison, WI, USA) and incubated on ice for 1 h. Samples were cleared by centrifugation for 10 min at 13,000g using an ultracentrifuge. Transfection efficiency was measured using a luminometer (TD20/20, Turner Design, Sunnyvale, CA, USA), and total protein content in the supernatants was determined by BCA assay (Promega).

### *In vivo* therapeutic effect

Rats were anesthetized with Zoletil 50 (10 mg/rat; Virbac. Carros, France), and laminectomy was performed at the T9 level via clip compression for 10 min. PLGA/DC-Chol nanospheres loaded with pSV-VEGF were immediately injected into the injured spinal cord with an insulin syringe. In other groups of rats, PLGA/pSV-VEGF, PEI/pSV-VEGF complex, naked pSV-VEGF, or PBS was injected into the injured spinal cord. Muscle and skin were closed with sutures. After surgery, sepazolin (50 mg/kg; Yuhan Corporation) and bupreenorphin (0.1mg/kg, Hanlim, Seoul, Korea) was administered for 7 days. Behavioral testing was performed using the Basso, Beattie, Bresnahan (BBB) functional scale every week for 6 weeks. Experimenters were blind to group identity of rats.

### Immunofluorescent staining

Two or four weeks after injury and injection, rats were sacrificed and perfused with saline containing 4% PFA (pH 7.4; Merck, Germany) to obtain spinal cord tissue. For histological analyses, spinal cords were dissected and cut into 10 μm sections. Sections were stained using fluorescently-tagged antibodies against neurofilament (NF; Abcam, Cambridge, MA), GFAP (Abcam), or MAP-2 (Abcam). After obtaining images of the sections, Metamorph software (Universal Imaging, West Chester, PA) was used to measure NF-positive areas. To analyze microvessels, sections were stained using antibodies against SM-α actin (Abcam) followed by FITC-conjugated anti-mouse IgG (Jackson ImmunoResearch Laboratories). Twelve section from each rat were randomly selected for quantification. Samples were analyzed using a laser confocal microscope (LSM 700; Zeiss, Oberkochen, Germany).

### Reverse transcription polymerase chain reaction (RT-PCR)

Total sample RNA from transfected cell (n = 6) or spinal cord (n = 5) was extracted using an RNA extraction kit (Qiagen, Valencia, California, USA). Isolated RNA was converted to complementary DNA (cDNA) using the AccuPower RTPReMix Kit (Bioneer Inc., Daejeon, South Korea). Synthesized cDNA was amplified by PCR using the following primers: luciferase 5′- CAA ATC ATT CCG GAT ACT GCG-3′ (forward), 5′- GAA TTA CAC GGC GAT CTT TCC -3′ (reverse) and GAPDH 5′- CAT GGT GGT GAA GAC GCC AG-3′ (forward), 5′-CCT CCT CAT TGA CCT CAA CT—3′ (reverse). PCR was carried out for 30 cycles of denaturation (94°C, 30 s), annealing (55°C, 30 s), and extension (72°C, 45 s), with a final extension at 72°C for 10 min. PCR products were visualized by electrophoresis on 1% (w/v) agarose gel with ethidium bromide staining and analyzed using a gel documentation system (MiniBIS Pro; Bio-imaging systems Ltd. Cambridge, UK).

### Statistical analysis

Quantitative data were expressed as mean ± standard deviation (SD). Differences between groups were analyzed by one-way analysis of variance (ANOVA). Newman-Keuls tests were used for post-hoc comparisons between subgroups. Statistical analysis was performed using Medcalc software. A *p*-value of less than 0.05 was considered statistically significant.

## Results

### Characterization of PLGA /DC-Chol nanospheres and their release *in vitro*

**Fig 1:**
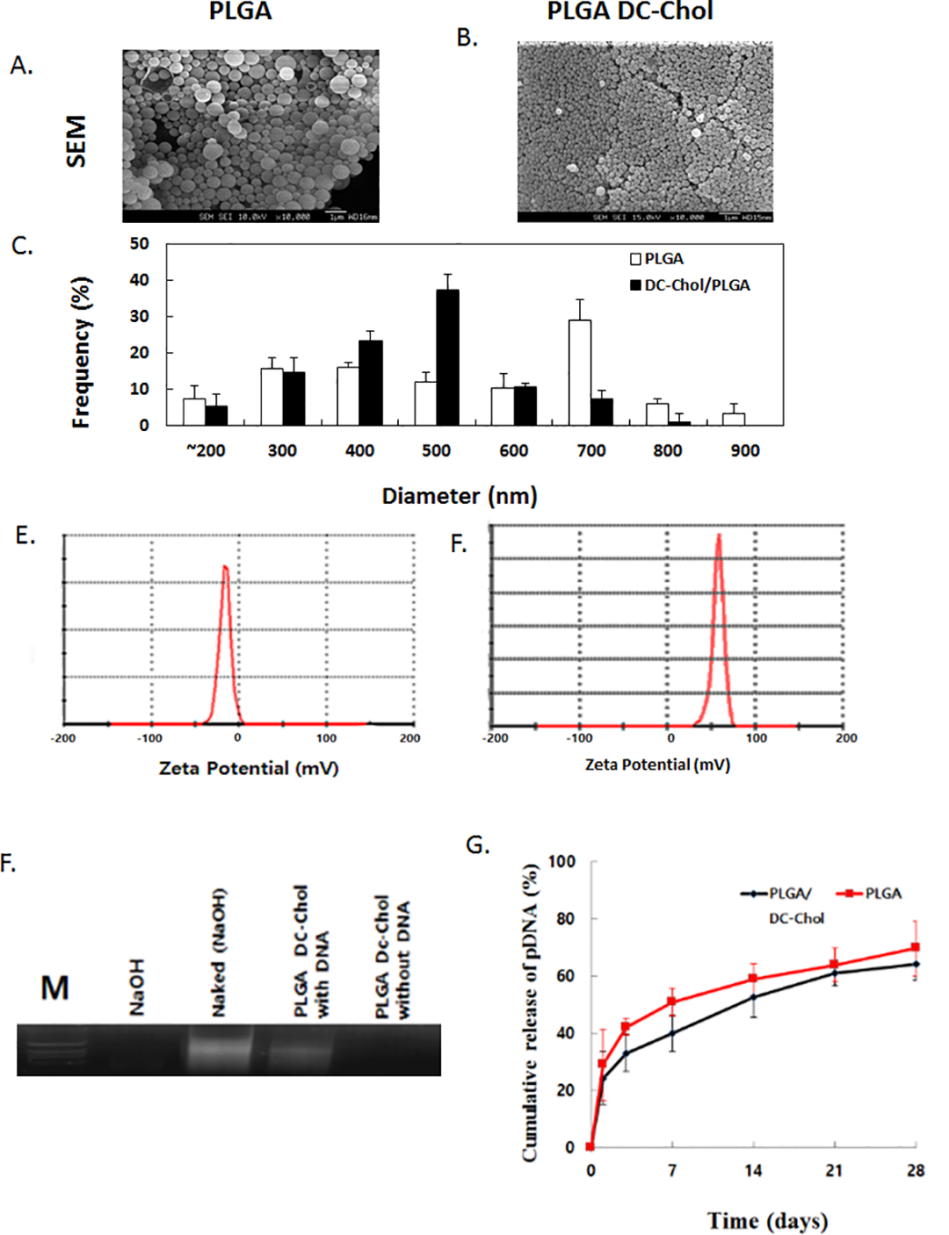
Characterization of nanospheres. SEM images of (A) PLGA and (B) PLGA/DC-Chol nanospheres. (C) Size distribution of nanospheres. Zeta potential (mV) of (D) PLGA and (E) PLGA/DC-Chol nanospheres. (F) Agarose gel retardation analysis. Agarose gel electrophoresis of pDNA released from PLGA and PLGA/DC-Chol nanospheres. (G) Cumulative release of pDNA from PLGA and PLGA/DC-Chol nanospheres.

Zeta potentials of nanospheres depended on their composition, with PLGA nanospheres containing the highest amount of DC-Chol undergoing a negative–to-positive inversion of zeta potential (Table 1). Scanning electron microphotographs showed that fabricated PLGA and PLGA/DC-Chol nanospheres were discrete spheres without aggregation and smooth in surface morphology (Fig 1A and 1B). PLGA/DC-Chol nanospheres were uniform in size. The average diameter of PLGA and PLGA/DC-Chol nanospheres was 619 ± 45.5 and 444 ± 21.1 nm, respectively (Fig 1C). The average zeta potential of PLGA and PLGA/DC-Chol nanoparticles was -34 ± 1.4 and 50.2 ± 8.7 mv, respectively (Fig 1D and 1E). The theoretical loading amount was approximately 58 mg pDNA/mg of PLGA nanospheres, and pDNA loading efficiency was 68.2% ± 12.3 (n = 10). Gel electrophoresis assay for pDNA purity and structural integrity before and after encapsulation by nanospheres revealed that the gel retardation pattern of pDNA extracted from nanospheres was similar to that of un-complexed pDNA (Fig 1F). In vitro pDNA release from PLGA and PLGA/DC-Chol nanospheres was sustained for at least 20 days (Fig 1G). The initial burst released approximately 35–40% of pDNA from nanospheres, after which pDNA continued to be released for over 20 days. The sustained pDNA release at later time points was due to the diffusion of DNA through the erosion of PLGA polymer.

**Table 1:** Physiochemical characterization of PLGA/DC-Chol nanospheres.

Formulation	DNA Loaded nanosphere	
PLGA: DC-Chol	Size (nm)	Zeta (mV)
100:0	619 ± 45.5	-34 ± 1.4
100:10	553 ± 37.4	-4 ± 2.1
100:20	444 ± 21.1	50.2 ± 2.7
100:40	432 ± 19.8	56.1 ± 1.2

### Cytotoxicity and the cellular uptake of nanospheres and cytotoxicity

To visualize nanosphere uptake by mNSCs, cells were incubated with PLGA or PLGA /DC-Chol nanospheres loaded with fluorescent dye for 6 or 24 h. After incubation, cells were washed with PBS to remove nanospheres on the cell surface so that only fluorescence from internalized particles was measured [24]. After 6 h, cytoplasmic fluorescence was observed for mNSCs treated with PLGA or PLGA/DC-Chol nanospheres (S1 Fig Top), with a higher intensity of fluorescence for mNSCs treated with PLGA/DC-Chol nanospheres. After 24 h, mNSCs treated with PLGA/DC-Chol nanospheres showed more intense fluorescence in the nuclei and cytoplasm (S1 Fig Bottom), whereas mNSCs treated with PLGA nanospheres showed less fluorescence.

**Fig 2:**
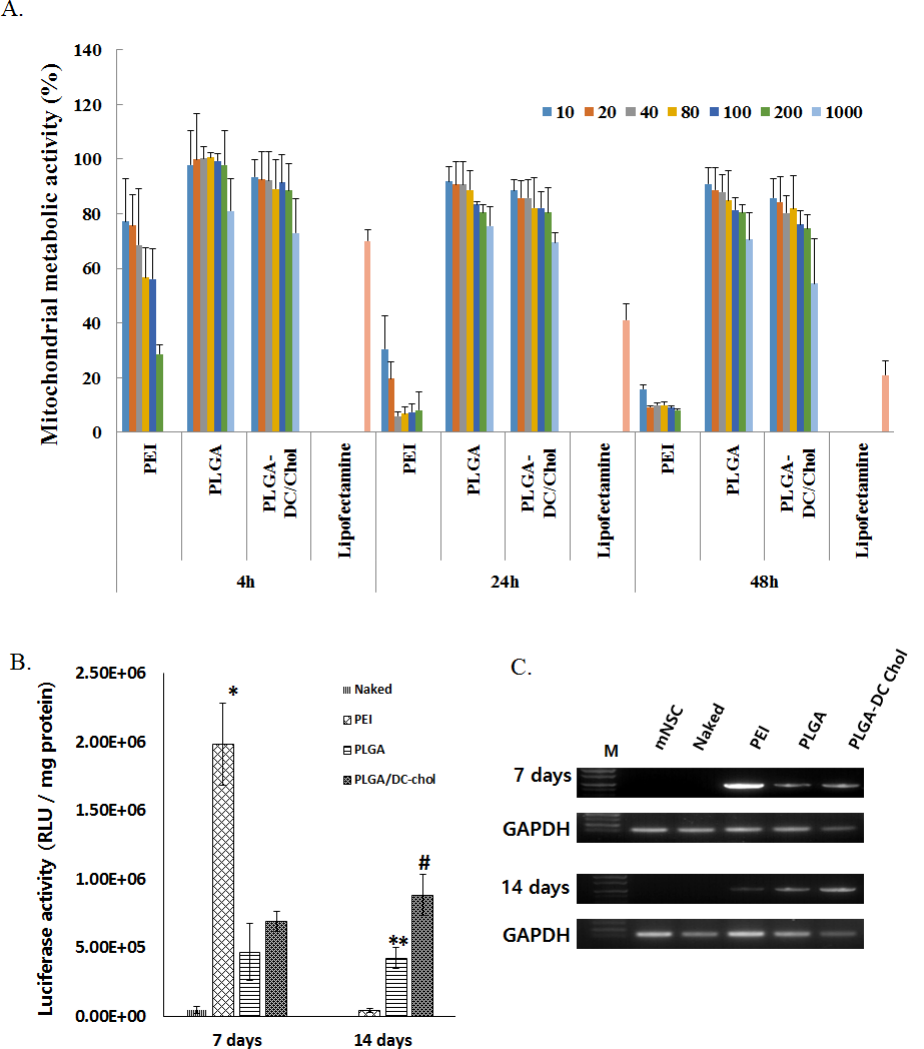
(A) Cytotoxicity of pDNA-loaded nanospheres and PEI/pDNA complexes. mNSCs were cultured for 4, 24, or 48 h with the indicated polymer concentrations, and mitochondrial metabolic activity in mNSCs was measured using the MTT assay. *p < 0*.*05* for nanospheres (PLGA/DC-Chol or PLGA) vs. PEI at all concentrations and time points (n = 5) except for 10 and 20 μg at 4 h. Transfection efficiency in cultured mNSCs (B, C). (B) Luciferase gene expression in mNSCs transfected with pSV-Luc-loaded PLGA nanospheres, PLGA/DC-Chol nanospheres, or PEI /pSV-Luc complex. *p < 0.05.PEI/pLuci compare with naked, PLGA and PLGA/DC-Chol at 7 days after transfection. *# p* < 0.05 PLGA/DC-Chol nanospheres compared with PLGA, PEI and naked at 14 days after transfection. **p < 0.05 PLGA nanospheres compared with PEI and naked at 14 days after transfection. (C) Luciferase mRNA expression 7 and 14 days after gene transfection.

To evaluate the cytotoxicity of nanospheres, mNSCs were treated with pSV-Luc-loaded PLGA nanospheres, PLGA/DC-Chol nanospheres, or PEI. PLGA and PLGA/DC-Chol nanospheres were significantly less cytotoxic than the PEI complex at various concentrations and incubation times (Fig 2A). PLGA/DC-Chol nanospheres produced similar levels of cytotoxicity as PLGA nanospheres at all concentrations. The PEI complex, however, was more cytotoxic at higher concentrations.

### *In vitro* transfection

Concentrations of neurospheres were normalized to their pDNA contents. Cultured mNSCs were incubated with 2 μg pSV-Luc/well, and expression of transgene delivered using PEI, PLGA nanospheres, or PLGA/DC-Chol nanospheres was compared. After 7 days, transgene expression was highest for PEI (Fig 2B and 2C). After 14 days, transgene expression decreased for PEI but increased for PLGA and PLGA/DC-Chol nanospheres, with a 2-fold higher transgene expression for PLGA/DC-Chol nanospheres compared with PLGA nanospheres. These results indicate that although PEI resulted in higher transgene expression after 7 days, DC-Chol enhanced the gene transfection efficiency of PLGA nanospheres 14 days after treatment *in vitro*.

### *In vivo* apoptosis in the rat spinal cord

**Fig 3:**
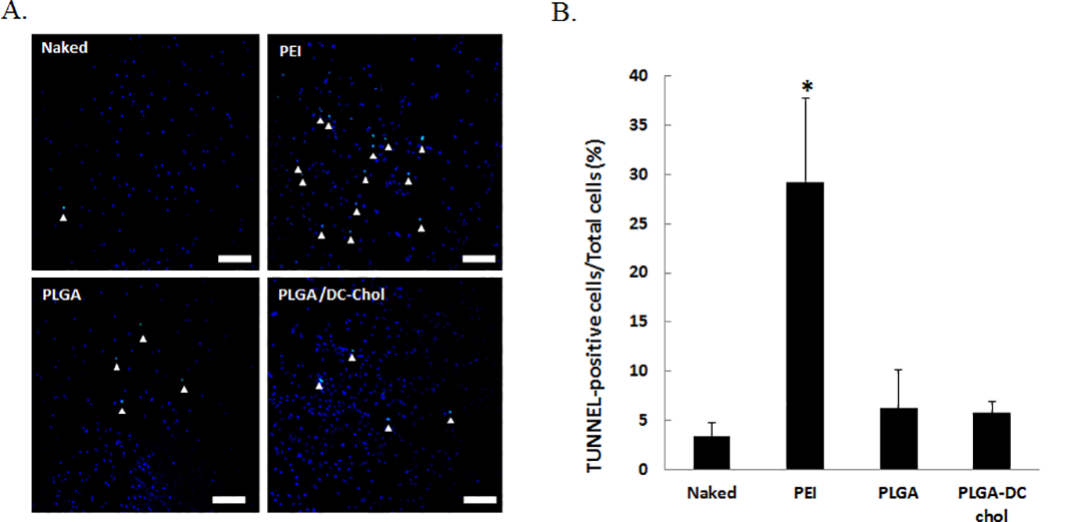
Apoptosis in the rat spinal cord. (A) Apoptotic activity at the injection site 24 h after injection. Nuclei were stained with DAPI (blue). Apoptosis-positive nuclei were stained with FITC (green) using a TUNEL staining method. Scale bars indicate 100 μm. (B) TUNEL-positive cell density at the injection site for naked pDNA, PEI/pDNA, PLGA/pDNA, and PLGA/DC-Chol/pDNA groups. *p < 0.05 compared with naked, PLGA nanospheres and PLGA/DC-Chol nanospheres

One day after injection of naked pSV-Luc, PEI/pSV-Luc complex, PLGA/pSV-Luc nanospheres, or PLGA/DC-Chol/pSV-Luc nanospheres into healthy rat spinal cords, apoptosis was assessed by TUNEL staining. There was significantly less TUNEL staining in PLGA/DC-Chol/pSV-Luc spinal cords compared with PEI/pSV-Luc spinal cords (Fig 3A). The density of apoptosis-positive cells in the spinal cord was significantly higher for the PEI/pSV-Luc group than the other groups (Fig 3B).

### *In vivo* gene expression in the rat spinal cord

**Fig 4:**
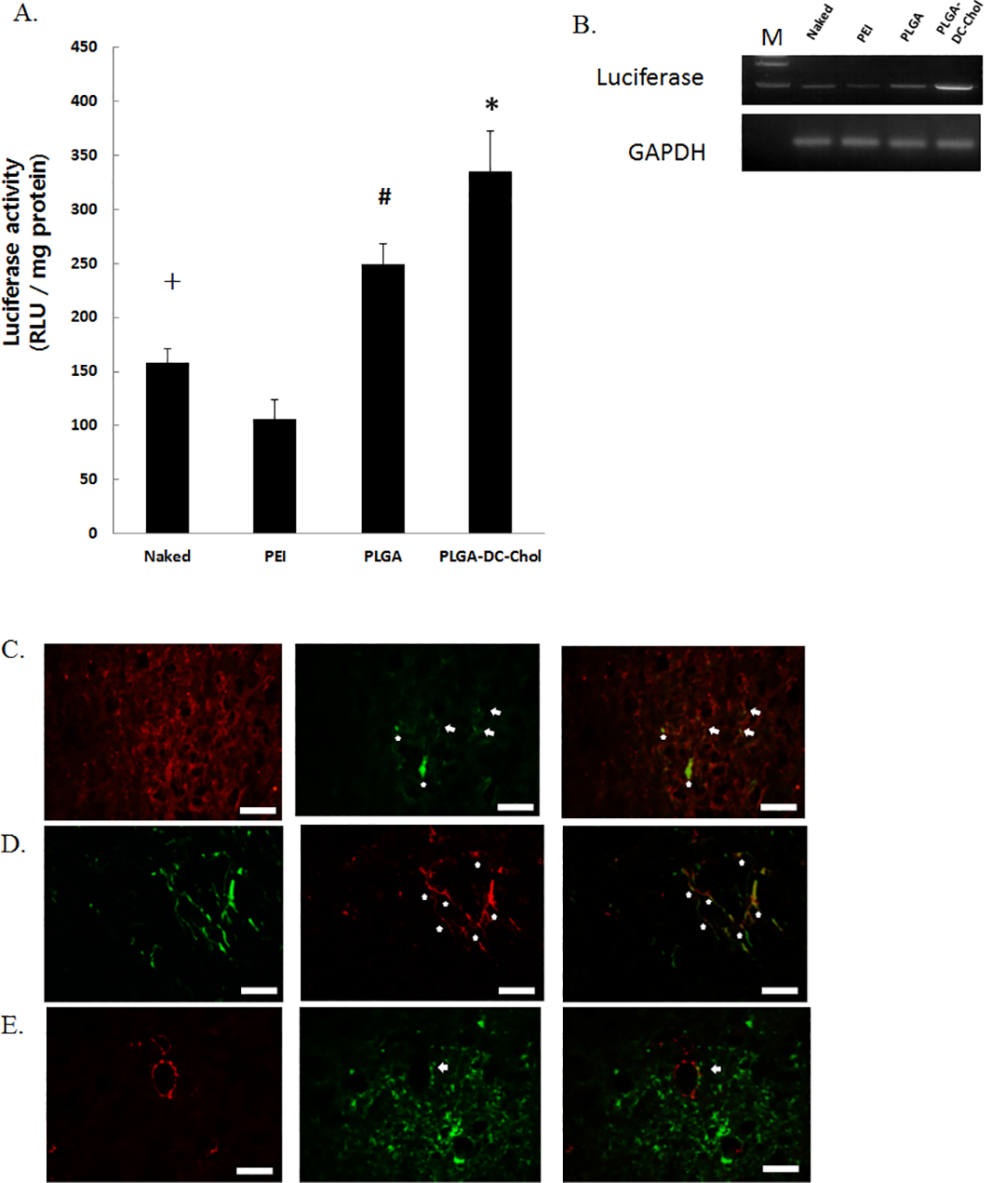
Gene expression in the rat spinal cord. (A, B) Luciferase activity in the rat spinal cord 14 days after injection. *p < 0.05 compared with naked pLuci and PLGA/pLuci. # *p* < 0.05 compared with naked and PEI/pDNA, + *p* < 0.05 compared with PEI/pDNA. Duble immunofluorescent staining for (C) beta III tublin (red), luciferease (green) and betaIII tublin/luciferase merged cells (yellow) (D) GFAP (green), luciferase (red) and GFAP/luciferase merged cells (yellow), (E) Smooth muscle α-actin (red), luciferase (green) and SM α-actin/luciferase merged cells(yellow) in spinal cord. The arrows indicate cells of luciferase expression and beta III tublin positive cells, GFAP positive cells or sm-α actin positive cells. Scale bars indicate 50 μm.

To evaluate the persistence of gene expression, naked pSV-Luc, PEI/pSV-Luc, PLGA/pSV-Luc nanospheres, or PLGA/DC-Chol/pSV-Luc nanospheres were injected into rat spinal cords. Fourteen days after injection, the PLGA/DC-Chol nanosphere group showed the highest level of luciferase expression (Fig 4A and 4B). We injected PLGA/DC-Chol/pSV-Luc nanospheres into the spinal cord to assay the distribution of luciferase expression following PLGA/DC-Chol delivery. Luciferase positive cells were observed near the injected site in the spinal cord. (Fig 4C, 4D and 4E). Luciferase expression was detected both in beta III tublin–positive neurons and GFAP-positive astrocytes in the site of the spinal cord injury (Fig 4C and 4D). Luciferase positive cells observed SM-α actin positive arteriole (Fig 4E). These results suggest that PLGA/DC-Chol/pSV-Luc nanospheres was delivery into the spinal cord, and that luciferase was expressed in both the neurons and astrocytes in the spinal cord.

### Angiogenesis and tissue regeneration

**Fig 5:**
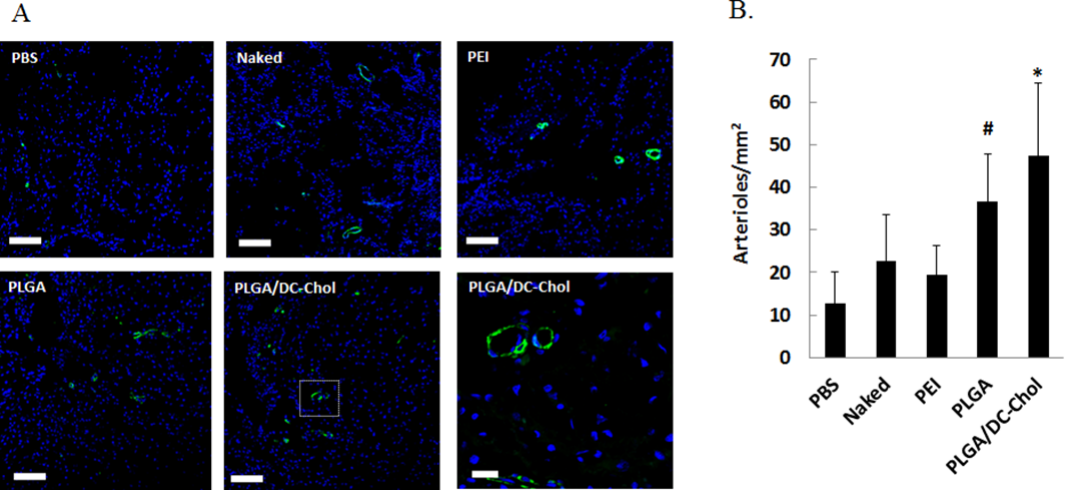
Angiogenesis after injection of VEGF-loaded PLGA/DC-Chol nanospheres. (A) Immunofluorescent staining for SM-α actin in injured spinal cords 4 weeks after injection. Scale bars indicate 100 μm at 100× and 20 μm at 400× (B) Quantification of arteriole density in injured spinal cords. *p < 0.05 vs. PBS, naked VEGF, or PEI/VEGF. #*p* < 0.05 vs. PBS.

Immunostaining for SM-α actin revealed more extensive angiogenesis in injured spinal cords of rats injected with VEGF-loaded PLGA/DC-Chol nanospheres compared with those of other groups 4 weeks after injection (Fig 5A). Quantification of arteriole density revealed that arteriole formation was significantly enhanced by injection of VEGF-loaded PLGA/DC-Chol nanospheres compared with injection of PBS, naked VEGF pDNA, or PEI/pVEGF complex (Fig 5B).

**Fig 6:**
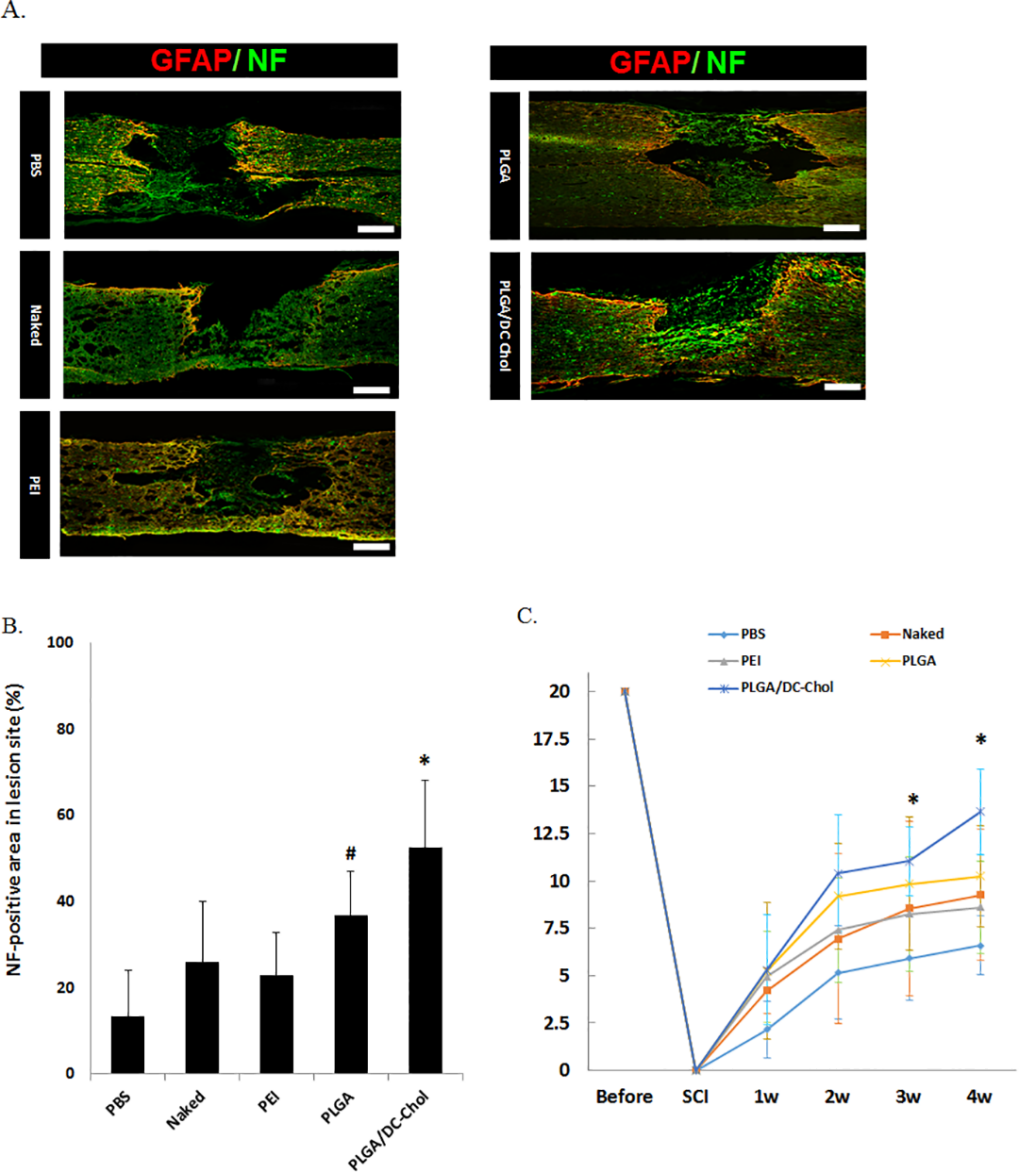
Axonal growth at lesion site and functional recovery 4 weeks after injury and injection. (A) Double immunostaining with GFAP (red) and NF (green). Scare bar indicates 100 μm. (B) Quantification of NF optical density of regenerated area. *p < 0.05 vs. PBS, naked VEGF, and PEI/VEGF. #p < 0.05 vs. PBS. (C) Effect of VEGF-loaded PLGA/DC-Chol nanospheres on recovery of locomotor function. BBB score was significantly greater in the PLGA/DC-Chol nanosphere group (n = 5) compared to PLGA/VEGF (n = 5), PEI/VEGF (n = 5), naked VEGF (n = 5), and PBS (n = 5) 3 and 4 weeks after injury. *p < 0.05 for PLGA/DC-Chol *vs*. PBS, naked VEGF, and PEI/VEGF groups.

Axonal growth was assessed by double staining with GFAP and NF 4 weeks after injection of PBS, naked pVEGF, PEI/pVEGF, PLGA/pVEGF nanospheres, or PLGA/DC-Chol/pVEGF nanospheres; areas positive for NF only were considered to indicate axonal regeneration [25,26]. Double staining with GFAP and NF revealed smaller lesion areas after injection of PLGA/DC-Chol nanospheres than after injection of PBS (Fig 6A). Areas positive for NF only in lesion sites were observed for all groups, although those in the PBS-treated group were disturbed due to linear glial scarring and large cavity formation. In the PLGA/DC-Chol nanosphere group, NF-positive axons were extensively present throughout the lesion site. Also, PLGA/pVEGF nanospheres was able to significantly increase NF-positive area compared to PBS treatment. Axonal regeneration in spinal cords injected with pVEGF-loaded PLGA/DC-Chol nanospheres was significantly greater than that in PBS, naked VEGF, and PEI/VEGF groups (Fig 6B), indicating that PLGA/DC-Chol nanospheres encapsulating VEGF pDNA could improve functional recovery as a result of enhanced angiogenesis and tissue restoration via axonal outgrowth in lesion areas.

### Recovery of locomotor function

Rats in the PLGA/DC-Chol/VEGF and PLGA/VEGF groups showed better recovery of locomotor function 2 weeks after injection compared with rats in the other groups (Fig 6C). Rats injected with PLGA/DC-Chol nanospheres encapsulating VEGF pDNA (n = 5, average score: 13.6±2.25) showed significantly greater functional recovery compared with rats injected with PLGA/VEGF nanospheres (n = 5, average score: 10.2±2.27), PEI/VEGF complex (n = 5, average score: 8.6±2.41), naked VEGF (n = 5, average score: 9.2±3.35), or PBS (n = 5, average score: 6.6±1.55) 4 weeks after injury. This enhanced functional recovery in rats treated with PLGA/DC-Chol nanospheres encapsulating VEGF pDNA may be a result of increased neovascularization and restoration of axonal outgrowth.

## Discussion

We investigated methods for preparing cationic PLGA nanospheres as non-viral vectors for gene delivery. We found that nanospheres made of PLGA/DC-Chol were less cytotoxic than PEI both *in vitro* and *in vivo*. PLGA/DC-Chol nanospheres were taken up by cells 6 and 24 h after injection, and injection of PLGA/DC-Chol nanospheres resulted in greater and longer-lasting gene expression compared with PEI both *in vitro* and *in vivo*. PLGA/DC-Chol nanospheres encapsulating VEGF pDNA enhanced axon growth and angiogenesis in injured rat spinal cords. VEGF-loaded PLGA/DC-Chol nanospheres not only improved the regeneration of injured spinal cord tissue but also promoted the recovery of locomotor activity in adult rats with spinal cord injury.

The use of PLGA/DC-Chol nanospheres as gene delivery vehicles provides several advantages over other methods, including increased cellular uptake and higher transgene expression. We found that the incorporation of greater amounts of the cationic lipid DC-Chol into the PLGA matrix significantly reduced particle size and increased nanosphere zeta potential (Table 1). This phenomenon of decreasing particle size with increasing amounts of cationic polymer has also been observed using DOPAP and PEI [17,27] and may be due to reduced interfacial tension between the particle surface and the aqueous medium. We also found that the zeta potential of PLGA nanospheres transitioned from negative to positive with increasing amounts of DC-Chol. These positive charges on nanosphere surfaces could enhance their adhesion to negatively charged cell surfaces, thereby facilitating their entry into cells by endocytosis[21]. Increasing amounts of DC-Chol, a cationic cholesterol derivative, in the PLGA matrix significantly increases the zeta potential of nanospheres, and pDNA is incorporated into the PLGA/DC-Chol nanospheres as the result of an electrostatic interaction between negatively charged pDNA with positively charged PLGA/DC-Chol nanoparticles [20,21]. The amount of cellular uptake of PLGA/DC-Chol nanospheres was higher than that of unmodified PLGA nanospheres (S1 Fig). Moreover, the encapsulation efficiency of pDNA and gene expression of PLGA/DC-Chol nanospheres was increased by the presence of DC-Chol (Fig 2B and 2C).

The use of PLGA/DC-Chol nanospheres allowed for long-term transgene expression, with the sustained release of pDNA from nanospheres for at least 14 days (Fig 2). We found that, 7 days after transfection, the level of pDNA gene expression was higher for PEI than for PLGA and PLGA/DC-Chol nanospheres. Fourteen days after transfection, however, this trend was reversed, likely due to sustained pDNA release from degraded PLGA via hydrolysis [14]. Previous studies have also showed sustained release of pDNA and gene expression from PLGA nanospheres [7,12]. This longer gene expression from PLGA nanospheres may be due to the protection of pDNA from nuclease degradation and the prolongation of pDNA half-life after cell uptake [12,28]. Furthermore, we found that DC-Chol modification enhanced gene expression from PLGA/DC-Chol nanospheres both *in vitro* and *in vivo* (Fig 2B and 2C). Also, PLGA DC-Chol nanospheres loaded with pDNA showed higher transgene expression than naked pDNA *in vivo*. Therefore, PLGA/DC-Chol nanospheres could be used as vectors for the sustained release of pDNA and the long-term expression of genes.

Compared to PEI, treatment with PLGA/DC-Chol nanospheres led to improved cell viability of mNSCs and rat spinal cord tissue. Our results show that DC-Chol-modified PLGA nanospheres have a better safety profile than PEI. Also, the positive charges of PLGA/DC-Chol nanospheres did not reduce cell viability or increase apoptosis compared with unmodified PLGA nanospheres (Figs 2A and 3). Liposomes and cationic polymer are major non-viral vectors that have been applied in various stages of clinical trials [19]. Although liposomes have advantages such as low immunogenicity and ease of preparation, their use is limited due serious cytotoxic side effects, low transfection efficiency, and instability in serum [29]. Also, although PEI, a cationic polymer, has been used as a gene delivery carrier because of its high transfection efficiency, its high cytotoxicity severely limits its application *in vivo* [30]. By contrast, PLGA is a biocompatible and biodegradable polymer that is approved for human applications. Also, several studies have implanted PLGA nerve conduit or PLGA scaffold for regeneration of spinal cord after injury and evaluated the interaction of host tissue [31–33]. Implantation of the PLGA scaffold with cells promoted long-term improvement in function [31,34]. Although degradation of PLGA is created an acidic environment, PLGA nanoparticles did not impact the proliferation of astrocytes or the survival of neuron in vitro [35]. In the present study, TUNEL analysis did not show significant evidence of tissue apoptosis caused by PLGA nanospheres. Our results show that DC-Chol-modified PLGA nanospheres have a better safety profile than PEI. Also, the positive charges of PLGA/DC-Chol nanospheres did not reduce cell viability or increase apoptosis compared with unmodified PLGA nanospheres (Figs 2A and 3).

Our findings indicate that nanospheres encapsulating VEGF pDNA could serve as efficient therapeutic vehicles for VEGF gene delivery to spinal cord injury, as their injection induced long-term VEGF expression and increased angiogenesis. Angiogenesis has been closely related function recover after CNS disease such as strock or SCI due to enough blood supply and oxygen supply for neural regeneration [36,37]. Increased angiogenesis after SCI induces endogenous recovery mechanisms, neuron survival and promote functional recovery [38]. Prior studies report that high VEGF expression and angiogenesis improves oxygen supply to the spinal cord after injury and increases axonal outgrowth and functional recovery [25,26]. Consistent with these previous studies, the present study confirms that nanospheres encapsulating VEGF pDNA can stimulate angiogenesis in the injured spinal cord and lead to recovery of locomotor function.

In summary, DC-Chol-modified PLGA nanospheres were less cytotoxic than PEI *in vitro* and *in vivo*. Also, VEGF gene delivery to the injured spinal cord using DC-Chol-modified PLGA nanospheres resulted in more angiogenesis and greater therapeutic effects 4 weeks after treatment compared with PBS or VEGF gene delivery via PEI or unmodified PLGA nanospheres. Further studies will be necessary to assess the clinical utility of this method, particularly those that are longer (i.e., more than 2 months) in duration. Additional studies should assess functional recovery using electrical measurements and VEGF effect post-injection of plasmid encapsulated PLGA/DC-Col nanospheres after spinal cord injury. Future studies will extend the capabilities of PLGA/DC-Chol nanospheres using the combined plasmid therapy such as BDNF and FGF in spinal cord injury models. Our findings suggest that DC-Chol-modified PLGA nanospheres could potentially serve as vehicles for gene delivery for spinal cord injury.

## Supporting Information

S1 Fig Cellular uptake of nanospheres. Cellular uptake of nanospheres. Confocal microscopic images showing cellular uptake of PLGA or PLGA/DC-Chol nanospheres tagged with 6-coumarin (green) by mNSCs after 6 h (Top) or 24 h (Bottom) of incubation. Nuclei were stained with DAPI (blue) Scale bar indicates 10 μm.
(DOCX)
