# Supplementary material for: Therapeutic Use of 3β-[N-(N′,N′-Dimethylaminoethane) Carbamoyl] Cholesterol-Modified PLGA Nanospheres as Gene Delivery Vehicles for Spinal Cord Injury
Source: PLoS One. 2016 Jan 29;11(1):e0147389. doi: 10.1371/journal.pone.0147389 (PMC4732605; doi:10.1371/journal.pone.0147389)

Supporting Information

S1 Fig. Cellular uptake of nanospheres. Confocal microscopic images showing cellular uptake of PLGA or PLGA/DC-Chol nanospheres tagged with 6-coumarin (green) by mNSCs after 6 h (Top) or 24 h (Bottom) of incubation. Nuclei were stained with DAPI (blue) Scale bar indicates 10 µm.

S1 Fig.


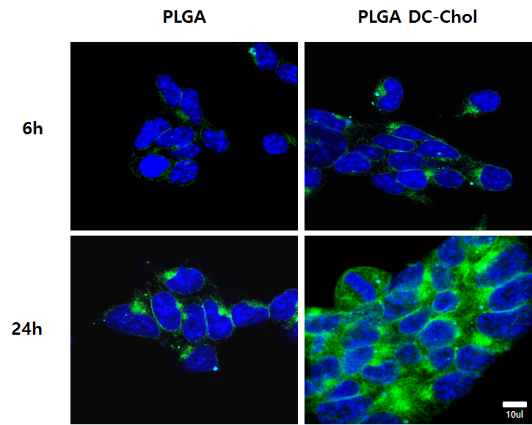

Supplement: S1 Fig — Cellular uptake of nanospheres. Confocal microscopic images showing cellular uptake of PLGA or PLGA/DC-Chol nanospheres tagged with 6-coumarin (green) by mNSCs after 6 h (Top) or 24 h (Bottom) of incubation. Nuclei were stained with DAPI (blue) Scale bar indicates 10 μm. (DOCX) [file pone.0147389.s001.docx]
